# 3-Methoxy­benzohydrazide

**DOI:** 10.1107/S1600536809036526

**Published:** 2009-09-16

**Authors:** Rifat Ara Jamal, Uzma Ashiq, Muhammad Nadeem Arshad, Zahida Tasneem Maqsood, Islam Ullah Khan

**Affiliations:** a Department of Chemistry, University of Karachi, Karachi 75270, Pakistan; b Department of Chemistry, Government College University, Lahore, Pakistan

## Abstract

The title compound, C_8_H_10_N_2_O_2_, crystallizes with two independent mol­ecules in an asymmetric unit. The structure is stabilized by nine inter­molecular N—H⋯N, N—H⋯O and C—H⋯O hydrogen bonds.

## Related literature

For related structures, see: Ashiq, Jamal *et al.* (2008 ▶, 2009*a* ▶,*b* ▶); Jamal *et al.* (2008 ▶, 2009 ▶); Kallel *et al.* (1992 ▶); Saraogi *et al.* (2002 ▶). For the biological activity of hydrazides, see: Ara *et al.* (2007 ▶); Ashiq, Ara *et al.* (2008 ▶).
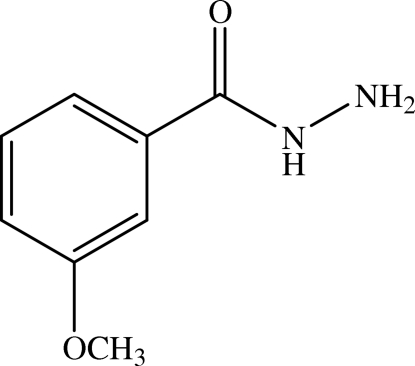


## Experimental

### 

#### Crystal data

C_8_H_10_N_2_O_2_
*M* *_r_* = 166.18
Monoclinic, 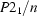

*a* = 13.1288 (8) Å
*b* = 7.5417 (4) Å
*c* = 17.0734 (10) Å
β = 92.857 (3)°
*V* = 1688.40 (17) Å^3^
*Z* = 8
Mo *K*α radiation
μ = 0.10 mm^−1^
*T* = 296 K
0.39 × 0.15 × 0.11 mm

#### Data collection

Bruker KAPPA APEXII CCD diffractometer
Absorption correction: multi-scan (*SADABS*; Bruker, 2005 ▶) *T*_min_ = 0.979, *T*_max_ = 0.990
18386 measured reflections
4200 independent reflections
1708 reflections with *I* > 2σ(*I*)
*R*_int_ = 0.066

#### Refinement

*R*[*F* ^2^ > 2σ(*F* ^2^)] = 0.049
*wR*(*F* ^2^) = 0.126
*S* = 0.95
4200 reflections
237 parameters
H atoms treated by a mixture of independent and constrained refinement
Δρ_max_ = 0.15 e Å^−3^
Δρ_min_ = −0.22 e Å^−3^

### 

Data collection: *APEX2* (Bruker, 2007 ▶); cell refinement: *SAINT* (Bruker, 2007 ▶); data reduction: *SAINT*; program(s) used to solve structure: *SHELXS97* (Sheldrick, 2008 ▶); program(s) used to refine structure: *SHELXL97* (Sheldrick, 2008 ▶); molecular graphics: *ORTEP-3 for Windows* (Farrugia, 1997 ▶); software used to prepare material for publication: *SHELXL97*.

## Supplementary Material

Crystal structure: contains datablocks I. DOI: 10.1107/S1600536809036526/pv2207sup1.cif

Structure factors: contains datablocks I. DOI: 10.1107/S1600536809036526/pv2207Isup2.hkl

Additional supplementary materials:  crystallographic information; 3D view; checkCIF report

## Figures and Tables

**Table 1:** Hydrogen-bond geometry (Å, °)

*D*—H⋯*A*	*D*—H	H⋯*A*	*D*⋯*A*	*D*—H⋯*A*
N11—H11*N*⋯N12^i^	0.91 (2)	2.12 (2)	2.958 (2)	153.3 (18)
N11—H11*N*⋯N11^i^	0.91 (2)	2.63 (2)	3.191 (2)	120.9 (13)
N12—H12*N*⋯O11^ii^	0.95 (2)	2.30 (2)	3.193 (2)	155.2 (18)
N12—H13*N*⋯N22^iii^	0.85 (2)	2.47 (2)	3.185 (3)	142.6 (19)
N21—H21*N*⋯O11^iv^	0.87 (2)	1.99 (2)	2.860 (2)	172 (2)
N22—H23*N*⋯O21^v^	0.88 (2)	2.14 (2)	3.007 (2)	168 (2)
C18—H18*C*⋯O21^vi^	0.96	2.58	3.498 (3)	160
C22—H22⋯O11^iv^	0.93	2.35	3.208 (3)	153
C26—H26⋯O21^vii^	0.93	2.56	3.439 (3)	157

Symmetry codes: (i) 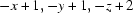; (ii) 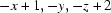; (iii) 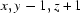; (iv) 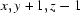; (v) 
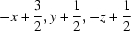
; (vi) 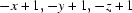; (vii) 
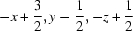
.

**Fig. 1.**
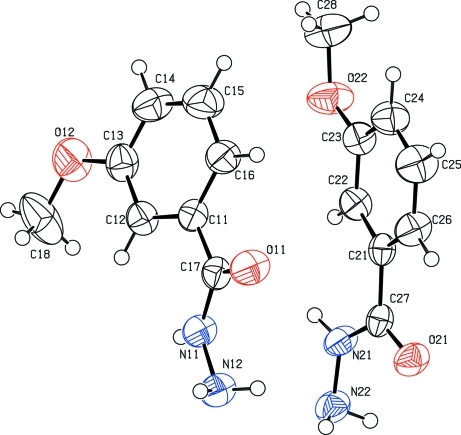
ORTEP diagram of the title compound with the ellipsoids drawn at the 50% probability level, showing the atomic labels.

**Fig. 2.**
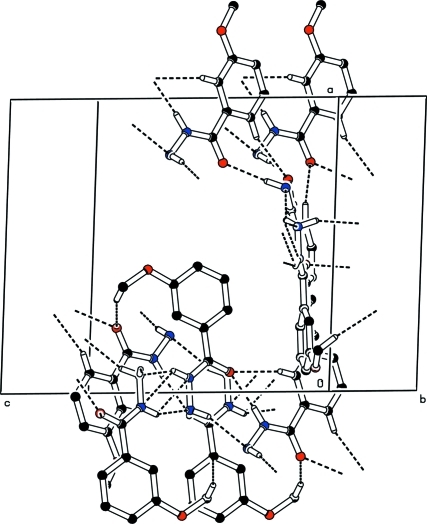
A unit cell packing diagram of (I) showing hydrogen bonds drawn by dashed lines. Hydrogen atoms not involved in H-bonding have been omitted.

## supplementary crystallographic information

### Comment

Hydrazides constitute an important class of biologically active organic compounds (Ashiq, Ara *et al.*, 2008; Ara *et al.*, 2007). In searching for biologically active compounds we have synthesized the title compound, (I), and report its crystal structure in this paper.

The asymmetric unit of the title compound contains two crystallographically unique molecules (Fig. 1). The bond distances and bond angles in (I) are similar to the corresponding distances and angles reported in the structures of benzhydrazide (Kallel *et al.*, 1992), *para*-chloro (Saraogi *et al.*, 2002), *para*-bromo (Ashiq, Jamal *et al.*, 2008),*para*-iodo (Jamal *et al.*, 2008), *para*-methoxy (Ashiq, Jamal *et al.*, 2009*a*), *para*-hydroxy (Jamal *et al.*, 2009) and *para*-phenylsemicarbazide (Ashiq, Jamal *et al.*, 2009*b*) analogues of (I).

In the crystal structure the molecules are held together by intermolecular hydrogen bonds of the types N—H···N, N—H···O and C—H···O (Table 1 and Fig. 2).

The title compound was found to be active against DPPH (1,1-diphenyl-2-picrylhydrazyl) radical scavenging and inactive against α-glucosidase enzyme (Ashiq, Ara *et al.*, 2008).

### Experimental

All reagent-grade chemicals were obtained from Aldrich and Sigma Chemical companies and were used without further purification. To a solution of ethyl-3-methoxybenzoate (3.6 g, 20 mmol) in 75 ml ethanol, hydrazine hydrate (5.0 ml, 100 mmol) was added. The mixture was refluxed for 5 h and a solid was obtained upon removal of the solvent by rotary evaporation. The resulting solid was washed with hexane to afford (I) (yield 79%). Crystal of the title compound suitable for X-ray crystallographic study were grown from a solution of methanol by slow evaporation at room temperature.

### Refinement

The hydrogen atoms bonded to N atoms were taken from fourier synthesis and their coordinates were allowed to refine. The H atoms bonded to aryl and methyl C-atoms were positioned geometrically, with C—H = 0.93 and 0.96 Å, respectively. The *U*_iso_(H) were 1.2 times *U*_eq_ of aryl C and N atoms and 1.5 times *U*_eq_ of methyl C atoms.

### Crystal data

**Table:**

C_8_H_10_N_2_O_2_	*F*(000) = 704
*M**_r_* = 166.18	*D*_x_ = 1.308 Mg m^−^^3^
Monoclinic, *P*2_1_/*n*	Mo *K*α radiation, λ = 0.71073 Å
Hall symbol: -P 2yn	Cell parameters from 2161 reflections
*a* = 13.1288 (8) Å	θ = 3.3–20.5°
*b* = 7.5417 (4) Å	µ = 0.10 mm^−^^1^
*c* = 17.0734 (10) Å	*T* = 296 K
β = 92.857 (3)°	Needle, colourless
*V* = 1688.40 (17) Å^3^	0.39 × 0.15 × 0.11 mm
*Z* = 8	

### Data collection

**Table:**

Bruker KAPPA APEXII CCD diffractometer	4200 independent reflections
Radiation source: fine-focus sealed tube	1708 reflections with *I* > 2σ(*I*)
graphite	*R*_int_ = 0.066
ω scans	θ_max_ = 28.3°, θ_min_ = 2.4°
Absorption correction: multi-scan (*SADABS*; Bruker, 2005)	*h* = −17→17
*T*_min_ = 0.979, *T*_max_ = 0.990	*k* = −9→10
18386 measured reflections	*l* = −22→22

### Refinement

**Table:**

Refinement on *F*^2^	Primary atom site location: structure-invariant direct methods
Least-squares matrix: full	Secondary atom site location: difference Fourier map
*R*[*F*^2^ > 2σ(*F*^2^)] = 0.049	Hydrogen site location: inferred from neighbouring sites
*wR*(*F*^2^) = 0.126	H atoms treated by a mixture of independent and constrained refinement
*S* = 0.95	*w* = 1/[σ^2^(*F*_o_^2^) + (0.0499*P*)^2^] where *P* = (*F*_o_^2^ + 2*F*_c_^2^)/3
4200 reflections	(Δ/σ)_max_ < 0.001
237 parameters	Δρ_max_ = 0.15 e Å^−^^3^
0 restraints	Δρ_min_ = −0.22 e Å^−^^3^

### Special details

**Table:**

Geometry. All e.s.d.'s (except the e.s.d. in the dihedral angle between two l.s. planes) are estimated using the full covariance matrix. The cell e.s.d.'s are taken into account individually in the estimation of e.s.d.'s in distances, angles and torsion angles; correlations between e.s.d.'s in cell parameters are only used when they are defined by crystal symmetry. An approximate (isotropic) treatment of cell e.s.d.'s is used for estimating e.s.d.'s involving l.s. planes.
Refinement. Refinement of *F*^2^ against ALL reflections. The weighted *R*-factor *wR* and goodness of fit *S* are based on *F*^2^, conventional *R*-factors *R* are based on *F*, with *F* set to zero for negative *F*^2^. The threshold expression of *F*^2^ > σ(*F*^2^) is used only for calculating *R*-factors(gt) *etc*. and is not relevant to the choice of reflections for refinement. *R*-factors based on *F*^2^ are statistically about twice as large as those based on *F*, and *R*- factors based on ALL data will be even larger.

### Fractional atomic coordinates and isotropic or equivalent isotropic displacement parameters (Å^2^)

**Table:**

	*x*	*y*	*z*	*U*_iso_*/*U*_eq_	
O11	0.43146 (11)	0.04300 (19)	1.07269 (9)	0.0607 (5)	
O12	0.08143 (12)	0.4522 (2)	0.92286 (10)	0.0767 (5)	
N11	0.45156 (14)	0.3134 (2)	1.02178 (10)	0.0459 (5)	
H11N	0.4261 (15)	0.422 (3)	1.0077 (12)	0.055*	
N12	0.55933 (15)	0.3117 (2)	1.02887 (13)	0.0525 (5)	
H12N	0.5835 (16)	0.216 (3)	0.9985 (12)	0.063*	
H13N	0.5782 (17)	0.271 (3)	1.0737 (13)	0.063*	
C11	0.28175 (15)	0.1986 (2)	1.03161 (12)	0.0406 (5)	
C12	0.23758 (16)	0.3243 (3)	0.98111 (12)	0.0470 (6)	
H12	0.2785	0.4009	0.9538	0.056*	
C13	0.13289 (18)	0.3352 (3)	0.97163 (13)	0.0532 (6)	
C14	0.07247 (18)	0.2229 (3)	1.01231 (15)	0.0646 (7)	
H14	0.0018	0.2306	1.0059	0.078*	
C15	0.11629 (19)	0.0994 (3)	1.06246 (15)	0.0676 (7)	
H15	0.0751	0.0245	1.0904	0.081*	
C16	0.22077 (18)	0.0856 (3)	1.07168 (13)	0.0545 (6)	
H16	0.2500	0.0001	1.1049	0.065*	
C17	0.39396 (16)	0.1793 (3)	1.04354 (12)	0.0415 (5)	
C18	0.1395 (2)	0.5781 (4)	0.88291 (17)	0.0981 (11)	
H18A	0.1783	0.6490	0.9204	0.147*	
H18B	0.0946	0.6531	0.8516	0.147*	
H18C	0.1850	0.5177	0.8496	0.147*	
O21	0.72052 (11)	0.73719 (17)	0.21668 (8)	0.0534 (4)	
O22	0.27188 (11)	0.6456 (2)	0.17438 (10)	0.0692 (5)	
N21	0.61560 (13)	0.9272 (2)	0.15351 (11)	0.0471 (5)	
H21N	0.5565 (16)	0.954 (3)	0.1308 (12)	0.057*	
N22	0.69253 (15)	1.0512 (3)	0.13927 (12)	0.0531 (6)	
H23N	0.7151 (16)	1.092 (3)	0.1850 (13)	0.064*	
H22N	0.7419 (17)	0.994 (3)	0.1205 (14)	0.064*	
C21	0.54632 (15)	0.6517 (2)	0.20011 (11)	0.0372 (5)	
C22	0.44617 (16)	0.7004 (3)	0.18400 (12)	0.0442 (5)	
H22	0.4313	0.8147	0.1665	0.053*	
C23	0.36779 (16)	0.5814 (3)	0.19354 (12)	0.0460 (5)	
C24	0.38825 (17)	0.4133 (3)	0.21999 (13)	0.0553 (6)	
H24	0.3357	0.3330	0.2268	0.066*	
C25	0.48845 (19)	0.3654 (3)	0.23633 (14)	0.0640 (7)	
H25	0.5030	0.2514	0.2543	0.077*	
C26	0.56714 (17)	0.4813 (3)	0.22674 (13)	0.0523 (6)	
H26	0.6341	0.4459	0.2380	0.063*	
C27	0.63403 (16)	0.7742 (3)	0.19120 (11)	0.0382 (5)	
C28	0.18800 (17)	0.5274 (3)	0.17719 (16)	0.0711 (8)	
H28A	0.2005	0.4247	0.1459	0.107*	
H28B	0.1272	0.5858	0.1571	0.107*	
H28C	0.1796	0.4919	0.2305	0.107*	

### Atomic displacement parameters (Å^2^)

**Table:**

	*U*^11^	*U*^22^	*U*^33^	*U*^12^	*U*^13^	*U*^23^
O11	0.0571 (10)	0.0390 (9)	0.0842 (12)	−0.0005 (8)	−0.0140 (8)	0.0216 (8)
O12	0.0586 (11)	0.0935 (13)	0.0778 (13)	0.0257 (10)	−0.0005 (9)	0.0264 (10)
N11	0.0468 (12)	0.0323 (10)	0.0577 (12)	0.0019 (9)	−0.0059 (9)	0.0088 (9)
N12	0.0491 (13)	0.0412 (11)	0.0660 (15)	0.0008 (9)	−0.0087 (10)	0.0088 (10)
C11	0.0475 (13)	0.0319 (11)	0.0418 (13)	0.0002 (10)	−0.0044 (10)	0.0006 (10)
C12	0.0478 (15)	0.0476 (13)	0.0459 (14)	0.0062 (11)	0.0042 (11)	0.0032 (11)
C13	0.0547 (16)	0.0549 (15)	0.0498 (15)	0.0110 (13)	−0.0008 (12)	0.0053 (12)
C14	0.0481 (15)	0.0648 (17)	0.0801 (19)	−0.0026 (13)	−0.0052 (14)	0.0020 (14)
C15	0.0629 (18)	0.0576 (17)	0.082 (2)	−0.0121 (13)	0.0019 (14)	0.0150 (14)
C16	0.0579 (16)	0.0431 (13)	0.0615 (16)	−0.0066 (12)	−0.0072 (12)	0.0119 (11)
C17	0.0529 (14)	0.0315 (12)	0.0396 (13)	−0.0005 (11)	−0.0035 (10)	0.0026 (10)
C18	0.091 (2)	0.115 (3)	0.091 (2)	0.0472 (19)	0.0296 (18)	0.0613 (19)
O21	0.0428 (9)	0.0515 (10)	0.0644 (11)	0.0025 (7)	−0.0106 (8)	0.0114 (7)
O22	0.0410 (10)	0.0611 (11)	0.1053 (14)	0.0010 (8)	0.0027 (9)	0.0147 (9)
N21	0.0419 (11)	0.0390 (11)	0.0589 (13)	−0.0055 (9)	−0.0130 (9)	0.0077 (9)
N22	0.0564 (14)	0.0431 (12)	0.0585 (14)	−0.0114 (10)	−0.0096 (11)	0.0018 (10)
C21	0.0444 (13)	0.0347 (12)	0.0323 (12)	0.0042 (10)	−0.0003 (9)	−0.0027 (9)
C22	0.0490 (14)	0.0359 (12)	0.0479 (14)	0.0050 (11)	0.0030 (11)	0.0044 (10)
C23	0.0449 (14)	0.0453 (13)	0.0481 (14)	0.0048 (11)	0.0042 (11)	−0.0009 (10)
C24	0.0577 (17)	0.0415 (14)	0.0666 (16)	−0.0048 (11)	0.0038 (13)	0.0016 (12)
C25	0.0660 (18)	0.0361 (13)	0.089 (2)	0.0054 (13)	−0.0042 (14)	0.0130 (12)
C26	0.0491 (14)	0.0396 (13)	0.0670 (16)	0.0040 (11)	−0.0073 (12)	0.0050 (11)
C27	0.0451 (14)	0.0350 (12)	0.0339 (12)	0.0035 (10)	−0.0039 (10)	−0.0040 (9)
C28	0.0483 (16)	0.0705 (18)	0.095 (2)	−0.0070 (13)	0.0064 (14)	−0.0227 (15)

### Geometric parameters (Å, °)

**Table:**

O11—C17	1.234 (2)	O21—C27	1.228 (2)
O12—C13	1.368 (3)	O22—C23	1.373 (2)
O12—C18	1.415 (3)	O22—C28	1.419 (2)
N11—C17	1.327 (2)	N21—C27	1.337 (2)
N11—N12	1.414 (2)	N21—N22	1.407 (2)
N11—H11N	0.91 (2)	N21—H21N	0.87 (2)
N12—H12N	0.95 (2)	N22—H23N	0.88 (2)
N12—H13N	0.85 (2)	N22—H22N	0.85 (2)
C11—C16	1.374 (3)	C21—C22	1.380 (3)
C11—C12	1.389 (3)	C21—C26	1.385 (3)
C11—C17	1.484 (3)	C21—C27	1.490 (3)
C12—C13	1.378 (3)	C22—C23	1.381 (3)
C12—H12	0.9300	C22—H22	0.9300
C13—C14	1.372 (3)	C23—C24	1.368 (3)
C14—C15	1.372 (3)	C24—C25	1.379 (3)
C14—H14	0.9300	C24—H24	0.9300
C15—C16	1.377 (3)	C25—C26	1.369 (3)
C15—H15	0.9300	C25—H25	0.9300
C16—H16	0.9300	C26—H26	0.9300
C18—H18A	0.9600	C28—H28A	0.9600
C18—H18B	0.9600	C28—H28B	0.9600
C18—H18C	0.9600	C28—H28C	0.9600
			
C13—O12—C18	117.70 (18)	C23—O22—C28	118.35 (18)
C17—N11—N12	123.42 (17)	C27—N21—N22	122.87 (18)
C17—N11—H11N	123.5 (13)	C27—N21—H21N	123.3 (13)
N12—N11—H11N	112.5 (13)	N22—N21—H21N	113.5 (13)
N11—N12—H12N	108.7 (13)	N21—N22—H23N	107.0 (15)
N11—N12—H13N	109.0 (15)	N21—N22—H22N	107.2 (15)
H12N—N12—H13N	97.3 (19)	H23N—N22—H22N	106 (2)
C16—C11—C12	119.8 (2)	C22—C21—C26	118.87 (19)
C16—C11—C17	118.03 (18)	C22—C21—C27	123.24 (18)
C12—C11—C17	122.19 (19)	C26—C21—C27	117.88 (18)
C13—C12—C11	119.8 (2)	C21—C22—C23	120.78 (19)
C13—C12—H12	120.1	C21—C22—H22	119.6
C11—C12—H12	120.1	C23—C22—H22	119.6
O12—C13—C14	115.2 (2)	C24—C23—O22	124.6 (2)
O12—C13—C12	124.7 (2)	C24—C23—C22	120.4 (2)
C14—C13—C12	120.1 (2)	O22—C23—C22	115.02 (19)
C15—C14—C13	120.0 (2)	C23—C24—C25	118.6 (2)
C15—C14—H14	120.0	C23—C24—H24	120.7
C13—C14—H14	120.0	C25—C24—H24	120.7
C14—C15—C16	120.5 (2)	C26—C25—C24	121.8 (2)
C14—C15—H15	119.8	C26—C25—H25	119.1
C16—C15—H15	119.8	C24—C25—H25	119.1
C11—C16—C15	119.9 (2)	C25—C26—C21	119.6 (2)
C11—C16—H16	120.1	C25—C26—H26	120.2
C15—C16—H16	120.1	C21—C26—H26	120.2
O11—C17—N11	121.8 (2)	O21—C27—N21	120.57 (19)
O11—C17—C11	120.67 (18)	O21—C27—C21	121.92 (18)
N11—C17—C11	117.55 (17)	N21—C27—C21	117.51 (18)
O12—C18—H18A	109.5	O22—C28—H28A	109.5
O12—C18—H18B	109.5	O22—C28—H28B	109.5
H18A—C18—H18B	109.5	H28A—C28—H28B	109.5
O12—C18—H18C	109.5	O22—C28—H28C	109.5
H18A—C18—H18C	109.5	H28A—C28—H28C	109.5
H18B—C18—H18C	109.5	H28B—C28—H28C	109.5
			
C16—C11—C12—C13	0.1 (3)	C26—C21—C22—C23	−0.5 (3)
C17—C11—C12—C13	179.32 (18)	C27—C21—C22—C23	−179.80 (18)
C18—O12—C13—C14	176.3 (2)	C28—O22—C23—C24	−4.2 (3)
C18—O12—C13—C12	−4.0 (3)	C28—O22—C23—C22	175.51 (19)
C11—C12—C13—O12	−179.2 (2)	C21—C22—C23—C24	0.7 (3)
C11—C12—C13—C14	0.4 (3)	C21—C22—C23—O22	−179.05 (17)
O12—C13—C14—C15	179.5 (2)	O22—C23—C24—C25	179.3 (2)
C12—C13—C14—C15	−0.1 (4)	C22—C23—C24—C25	−0.4 (3)
C13—C14—C15—C16	−0.8 (4)	C23—C24—C25—C26	0.0 (3)
C12—C11—C16—C15	−1.0 (3)	C24—C25—C26—C21	0.1 (4)
C17—C11—C16—C15	179.8 (2)	C22—C21—C26—C25	0.1 (3)
C14—C15—C16—C11	1.3 (4)	C27—C21—C26—C25	179.42 (19)
N12—N11—C17—O11	0.9 (3)	N22—N21—C27—O21	1.5 (3)
N12—N11—C17—C11	−179.15 (17)	N22—N21—C27—C21	−178.00 (18)
C16—C11—C17—O11	16.9 (3)	C22—C21—C27—O21	168.96 (19)
C12—C11—C17—O11	−162.3 (2)	C26—C21—C27—O21	−10.3 (3)
C16—C11—C17—N11	−163.0 (2)	C22—C21—C27—N21	−11.6 (3)
C12—C11—C17—N11	17.8 (3)	C26—C21—C27—N21	169.15 (18)

### Hydrogen-bond geometry (Å, °)

**Table:**

*D*—H···*A*	*D*—H	H···*A*	*D*···*A*	*D*—H···*A*
N11—H11N···N12^i^	0.91 (2)	2.12 (2)	2.958 (2)	153.3 (18)
N11—H11N···N11^i^	0.91 (2)	2.63 (2)	3.191 (2)	120.9 (13)
N12—H12N···O11^ii^	0.95 (2)	2.30 (2)	3.193 (2)	155.2 (18)
N12—H13N···N22^iii^	0.85 (2)	2.47 (2)	3.185 (3)	142.6 (19)
N21—H21N···O11^iv^	0.87 (2)	1.99 (2)	2.860 (2)	172 (2)
N22—H23N···O21^v^	0.88 (2)	2.14 (2)	3.007 (2)	168 (2)
C18—H18C···O21^vi^	0.96	2.58	3.498 (3)	160
C22—H22···O11^iv^	0.93	2.35	3.208 (3)	153
C26—H26···O21^vii^	0.93	2.56	3.439 (3)	157

Symmetry codes: (i) −*x*+1, −*y*+1, −*z*+2; (ii) −*x*+1, −*y*, −*z*+2; (iii) *x*, *y*−1, *z*+1; (iv) *x*, *y*+1, *z*−1; (v) −*x*+3/2, *y*+1/2, −*z*+1/2; (vi) −*x*+1, −*y*+1, −*z*+1; (vii) −*x*+3/2, *y*−1/2, −*z*+1/2.
